# Hippocampal Subfield Volumes, Depression and Cognitive Decline in Alzheimer's Disease: A Longitudinal 7T Study

**DOI:** 10.1002/alz70862_110055

**Published:** 2025-12-23

**Authors:** Beili Shao, Oluwatobi F Adeyemi, Penny Gowland, Richard Bowtell, Olivier Mougin, Akram A. Hosseini

**Affiliations:** ^1^ School of Medicine, University of Nottingham, Nottingham, Nottinghamshire UK; ^2^ Academic Neurology, Nottingham University Hospitals NHS Trust, Queen's Medical Centre, Nottingham, Nottinghamshire UK; ^3^ Sir Peter Mansfield Imaging Centre, University of Nottingham, Nottingham, Nottinghamshire UK

## Abstract

**Background:**

Alzheimer's Disease (AD) is a neurodegenerative disorder characterized by progressive neuropathological changes, including significant hippocampal atrophy. Depression is a common co‐morbidity in AD, and its impact on disease progression remains an area of active research. This study aimed to investigate the prognostic value of 7T‐MRI‐based measurements of hippocampal subfield volumes for predicting cognitive decline over a year. We explored further the relationship between hippocampal subfield volumes and depression.

**Method:**

7T‐MRI‐derived hippocampal subfield volumes and clinical data from individuals diagnosed with amyloidβ status AD (*n* = 31) and HC (*n* = 26) participants were analysed through the Brain Iron Toxicity and Neurodegeneration (BITaN, IRAS 276174) study. Participants underwent an initial evaluation that included a 7T MRI scan and cognitive testing, followed by a subsequent cognitive assessment one year later. Correlations between hippocampal subfield volumes and depression score were assessed at the baseline. Predictive analyses were conducted to determine the extent to which baseline hippocampal subfield volumes could explain variations in clinical dementia rating (CDR) score decline.

**Result:**

In the AD group, significant negative correlations were observed between depression scores and hippocampal subfield volumes at the baseline, particularly in regions linked to emotional regulation such as the dentate gyrus (*r* = ‐0.35, *p* < 0.05) and subiculum (*r* = ‐0.31, *p* < 0.05). The average increase of CDR score after 1 year was 2.08±0.83 in the AD group (*p* <0.05 compared to HC group). Significant correlations were observed between the volume of certain hippocampal subfields (entorhinal cortex, subiculum, cornu ammonis 1 and dentate gyrus) at baseline and worsening CDR decline in a year, suggesting their potential as predictors of cognitive decline (*p* < 0.05). Interestingly, in the HC group, no significant correlation was found between depression and hippocampal subfield volumes. These findings highlight a differential role of hippocampal subfields in pathological versus non‐pathological aging processes.

**Conclusion:**

The study underscores the importance of hippocampal subfield volumes as markers of cognitive decline in AD. While depression correlates with subfield volumes in AD, this relationship is absent in healthy controls, suggesting distinct neurobiological underpinnings. These findings provide insights into potential targets for early intervention and highlight the need for further longitudinal studies.

